# Lupeol Is One of Active Components in the Extract of *Chrysanthemum indicum* Linne That Inhibits LMP1-Induced NF-κB Activation

**DOI:** 10.1371/journal.pone.0082688

**Published:** 2013-11-26

**Authors:** Se Chan Kang, Sue Yeon Lim, Yoon-Jae Song

**Affiliations:** Department of Life Science, Gachon University, Seongnam-Si, Kyeonggi-Do, Republic of Korea; Georgetown University, United States of America

## Abstract

We have previously reported that seventy percent ethanol extract of *Chrysanthemum indicum* Linne (CIE) strongly reduces Epstein-Barr virus (EBV)-transformed lymphoblastoid cell line (LCL) survival by inhibiting virus-encoded latent infection membrane protein 1 (LMP1)-induced NF-κB activation. To identify an active compound(s) in CIE that inhibits LMP1-induced NF-κB activation, activity-guided fractionation was employed. The CH_2_Cl_2_ fraction of CIE strongly reduced LMP1-induced NF-κB activation and LCL viability with relatively low cytotoxic effects on primary human foreskin fibroblast (HFF), HeLa or Burkitt’s lymphoma (BL41) cells. Furthermore, lupeol, a pentacyclic triterpene, was identified in the CH_2_Cl_2_ fraction of CIE to attenuate LMP1-induced NF-κB activation and LCL viability. This study demonstrates that lupeol is one of active compounds in the CH_2_Cl_2_ fraction of CIE that inhibits LMP1-induced NF-κB activation and reduces NF-κB-dependent LCL viability.

## Introduction

The NF-κB family of transcription factors plays an important role in tumoirgenesis, and aberrant NF-κB activation is a hallmark of many epithelial and lymphoid-derived cancers [1,2]. NF-κB promotes tumorigenesis by inducing expression of genes involved in cell proliferation, survival, tumor promotion, immortalization, angiogenesis and metastasis [1–3]. In addition, NF-κB is a critical regulator of inflammation and promotes inflammation-associated cancers [4].

The mammalian NF-κB family consists of RelA (p65), RelB, c-Rel, p105/p50 (NF-κB1) and p100/p52 (NF-κB2) [5,6]. In response to extracellular or intracellular stimuli including proinflammatory cytokines, tumor promoters, viral and bacterial infection or DNA damage, homo- or hetero-dimers of NF-κB family members are activated to transactivate various genes [3]. The inhibitor of κB (IκB) kinase (IKK) complex which is composed of two catalytic subunits, IKKα and β, and a regulatory subunit, IKKγ (or NEMO) is a key regulator of NF-κB activation. There are two major signaling pathways leading to NF-κB activation: the IKKβ- and IKKγ-dependent canonical NF-κB pathway and the IKKα-dependent non-canonical (or alternative) NF-κB pathway [5,6].

A canonical NF-κB pathway involves the heterodimeric p65/p50 complexes that are sequestered in the cytoplasm by IκB proteins. In response to various stimuli including pro-inflammatory cytokines, TNF-α and IL-1, and lipopolysaccharide (LPS), IκB proteins are phosphorylated by IKKβ and degraded by the ubiquitin-proteasome pathway allowing nuclear translocation of the p65/p50 complexes. The non-canonical NF-κB pathway which is utilized by lymphotoxin β (LT- β), B cell-activating factor of the TNF family (BAFF) and CD40 involves NF-κB inducing kinase (NIK)- and IKKα-mediated proteolytic processing of p100 into p52 and nuclear translocation of the RelB/p52 complexes [5,6].

Epstein-Barr virus (EBV) belongs to the human γ-herpesvirus family, and latent infection of EBV is causally associated with human lymphoid and epithelial malignancies including Burkitt’s lymphoma, T-cell lymphoma, Hodgkin’s disease, lymphoproliferative disease and nasopharyngeal carcinoma (NPC) [7,8]. *In vitro*, EBV latently infects primary B lymphocytes and transforms these cells into proliferating lymphoblastoid cell lines (LCLs). The EBV encoded Latent infection Membrane Protein 1 (LMP1) which is expressed in most EBV-associated cancers is essential for EBV-infected primary B lymphocytes conversion to LCLs [7,8]. LMP1 constitutively activate both the non-canonical and the canonical NF-κB pathways through two C-terminal cytoplasmic domains referred to as C-terminal Activation Regions 1 and 2 (CTAR1 and 2), respectively, and LMP1-induced NF-κB activation is critical for EBV-transformed LCL survival [9–18].

We have previously reported that *Chrysanthemum indicum* Linne extract (CIE) inhibits LMP1-induced NF-κB activation and LCL viability without exhibiting any adverse effect on the viability of cells whose survival is independent of NF-κB activation [19]. Therefore, in this study, we have expanded our investigation to identify an active compound(s) in CIE that inhibits LMP1-induced NF-κB activation and LCL viability by using activity-guided fractionation.

## Results

### The effect of CIE fractions on LMP1-induced NF-κB activation

**Figure 1:**
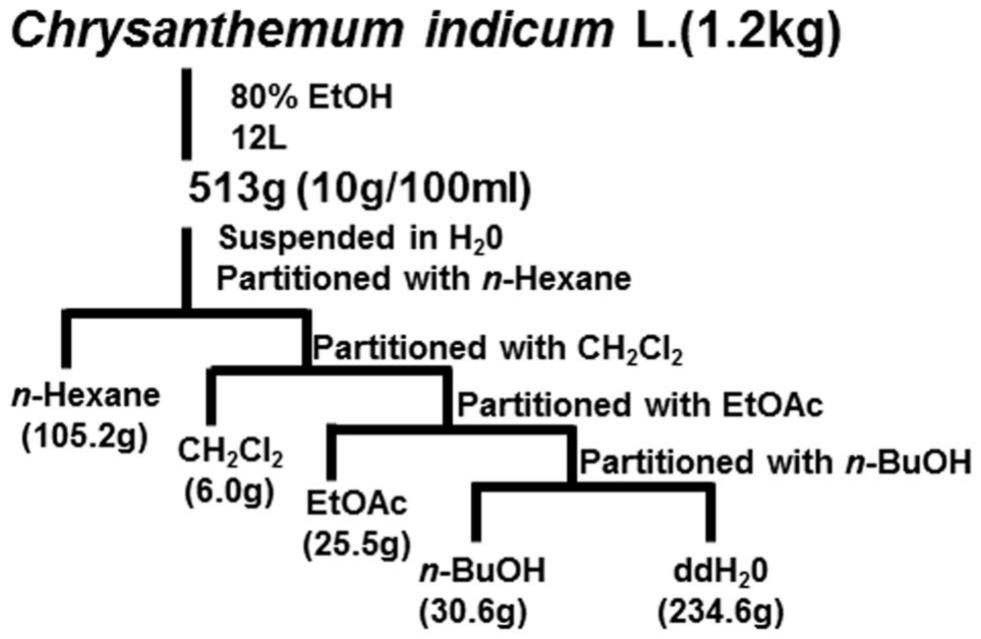
Fractionation scheme for the CIE.

**Figure 2:**
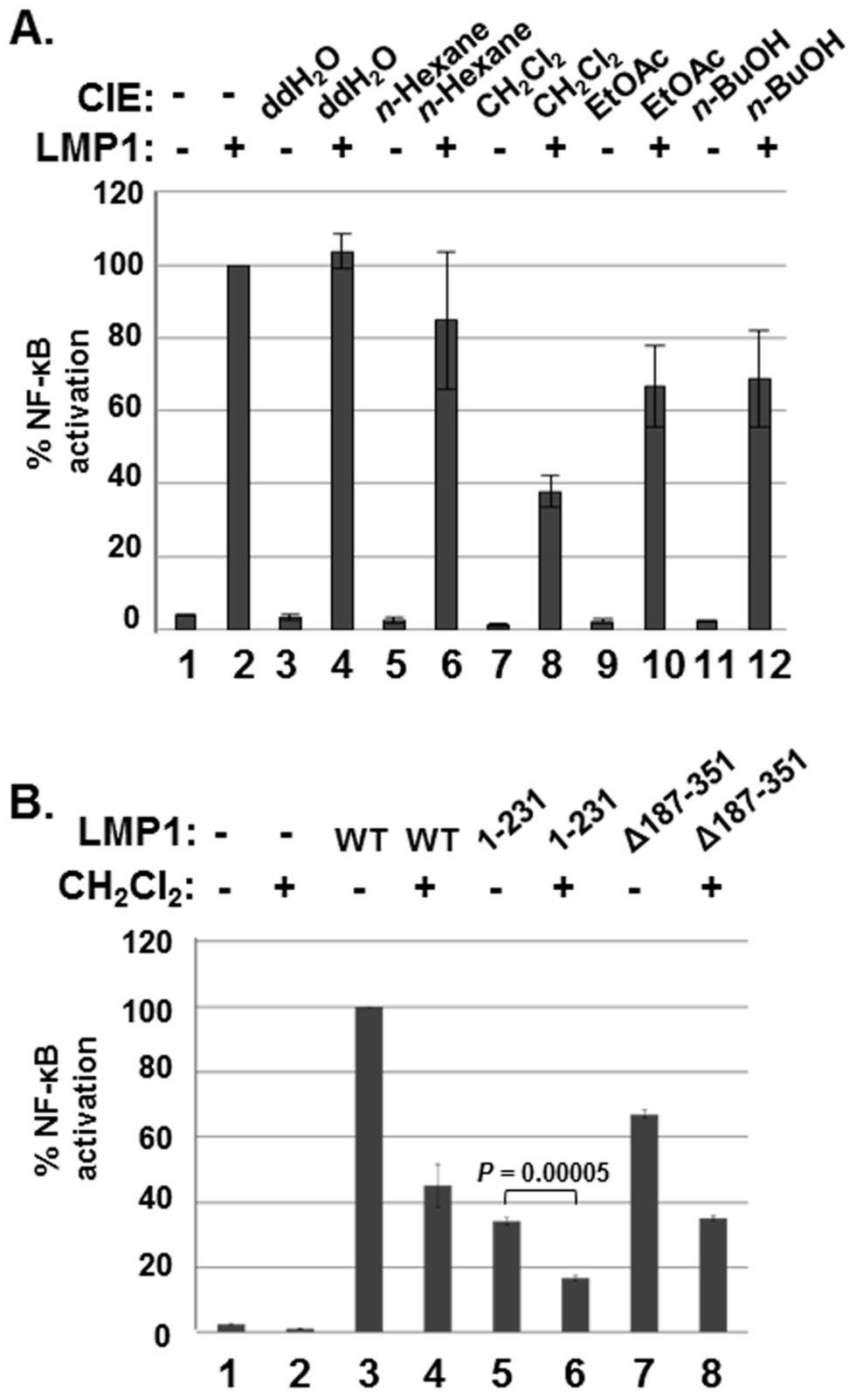
The CH_2_Cl_2_ fraction of CIE inhibits LMP1-induced NF-κB activation. (A) HEK293 cells were co-transfected with pSG5 (lanes 1, 3, 5, 7, 9, and 11) or pSG5-FLAG-LMP1 (lanes 2, 4, 6, 8, 10 and 12) plus NF-κB dependent firefly luciferase and control *Renilla* luciferase plasmids. Cells were treated with DMSO (lanes 1 and 2) or ddH_2_O (lanes 3 and 4), *n*-Hexane (lanes 5 and 6), CH_2_Cl_2_ (lanes 7 and 8), EtOAc (lanes 9 and 10) or *n*-BuOH (lanes 11 and 12) fraction of CIE at 100µg/ml, and luciferase activity was measured using a dual luciferase assay system. (B) HEK293 cells were co-transfected with pSG5 (lanes 1 and 2), pSG5-FLAG-LMP1 WT (lanes 3 and 4), pSG5-FLAG-LMP1 1-231 (lanes 5 and 6) or pSG5-FLAG-LMP1 Δ187-351 (lanes 7 and 8) plus NF-κB dependent firefly luciferase and control *Renilla* luciferase plasmids. Cells were treated with either DMSO (lanes 1, 3, 5 and 7) or the CH_2_Cl_2_ fraction of CIE (lanes 2, 4, 6 and 8) at 100µg/ml, and luciferase activity was measured using a dual luciferase assay system. NF-κB dependent luciferase activity was expressed in RLU by normalizing firefly luciferase activity with constitutive *Renilla* luciferase activity. To calculate relative luciferase activity, LMP1-induced luciferase activities in the presence of DMSO was set 100%. Significant difference between lanes 5 and 6 was determined by the P value of a two-sample *t* test (*P* < 0.0001). Luciferase data shown here represent three independent experiments. (RLU, relative luciferase unit).

To determine active constituents that inhibit LMP1-induced NF-κB activation, CIE was fractionated by sequential solvent extractions (Figure 1). Inhibitory activity of each fraction against LMP1-induced NF-κB activation was determined by using NF-κB-dependent luciferase reporter assays. Among the five tested fractions, the CH_2_Cl_2_ fraction reduced LMP1-induced NF-κB activation by 62% (Figure 2A, compare lane 8 with lane 2). The *n*-Hexane, EtOAc or *n*-BuOH fraction also reduced LMP1-induced NF-κB activation by 18%, 35% or 31%, respectively (Figure 2A, compare lanes 6, 10, 12 with lane 2). 

The effect of the CH_2_Cl_2_ fraction of CIE on LMP1 CTAR1- or CTAR2-induced NF-κB activation was further determined by using LMP1 mutants deleted for CTAR2 (aa 1-231) or CTAR1 (Δ187-351) (Figure 2B). The CH_2_Cl_2_ fraction of CIE reduced LMP1 CTAR1- or CTAR2-induced NF-κB activation by 51 or 54%, respectively (Figure 2B, compare lanes 6 and 8 with lanes 5 and 7). Taken together, comparing to other fractions, the CH_2_Cl_2_ fraction significantly reduced LMP1-induced NF-κB activation. Thus, the CH_2_Cl_2_ fraction of CIE may contain an active compound(s) that inhibits LMP1-induced NF-κB activation.

### The effect of the CH_2_Cl_2_ fraction of CIE on IKK activation

**Figure 3:**
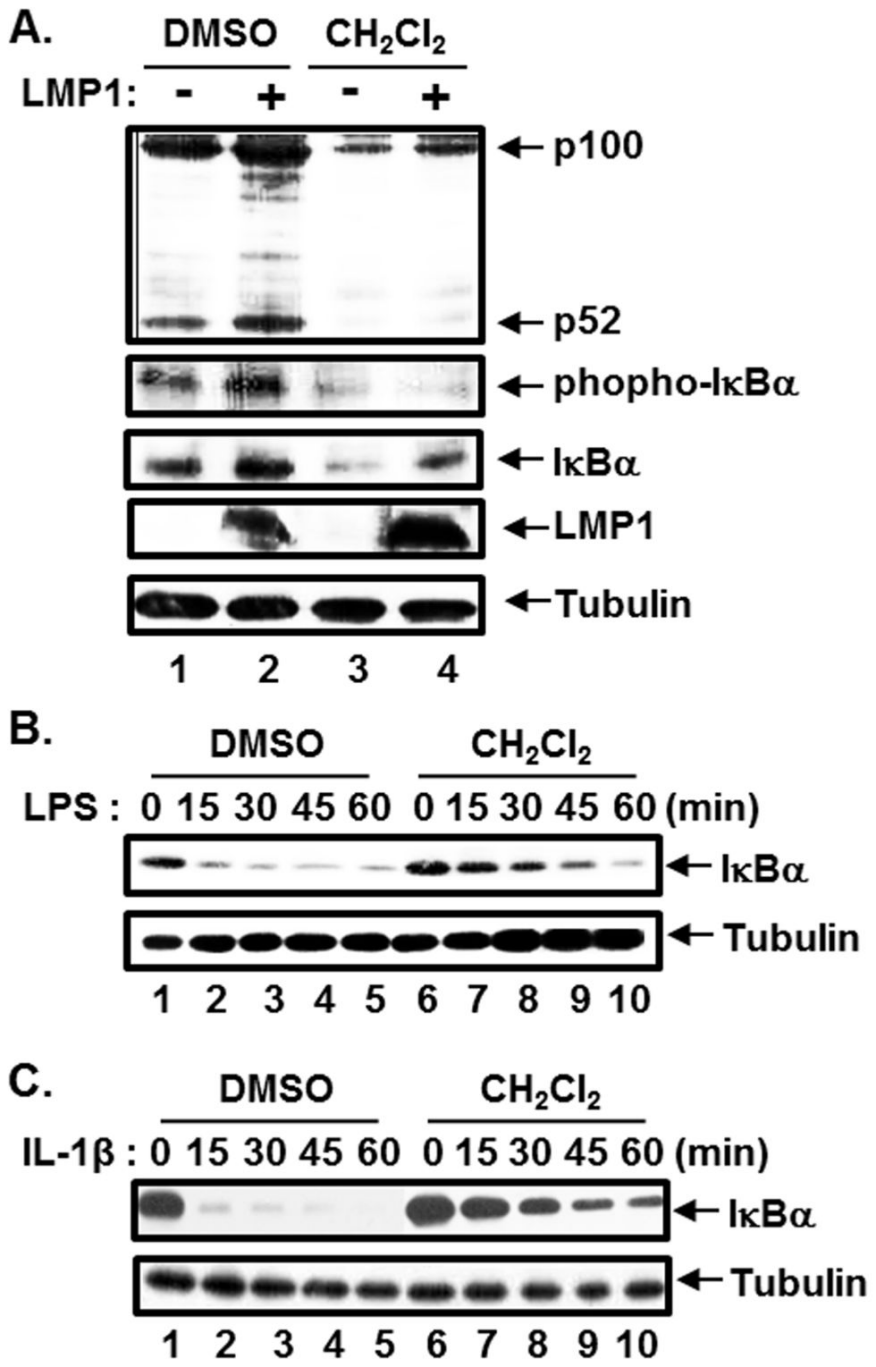
The CH_2_Cl_2_ fraction of CIE inhibits IKK activation. (A) Parental BL41 cells and their counterparts expressing LMP1 were treated with either DMSO or CH_2_Cl_2_ fraction of CIE at 100µg/ml for 24hr, and equal amounts of cell extracts were subjected to Western blot analysis with anti-p100/p52, anti-phopho-IκBα, anti-IκBα, anti-LMP1 or anti-tubulin antibody. (B) Raw 264.7 cells were pre-treated with either DMSO or CH_2_Cl_2_ fraction of CIE at 100µg/ml for 3hr and stimulated with LPS at 1µg/ml. At 0, 15, 30, 45 and 60 min after LPS treatment, equal amounts of cell extracts were subjected to Western blot analysis with anti-IκBα or anti-tubulin antibody. (C) HeLa cells were pre-treated with either DMSO or CH_2_Cl_2_ fraction of CIE at 100µg/ml for 3hr and stimulated with IL-1β at 20ng/ml. At 0, 15, 30, 45 and 60 min after IL-1β treatment, equal amounts of cell extracts were subjected to Western blot analysis with anti-IκBα or anti-tubulin antibody.

Since LMP1 activates both non-canonical and canonical NF-κB pathways through CTAR1 and CTAR2, respectively, the effect of the CH_2_Cl_2_ fraction of CIE on LMP1-induced IKKα or IKKβ activation was further investigated (Figure 3A). BL41 cells or their LMP1 expressing counterparts were treated with either DMSO or the CH_2_Cl_2_ fraction for 24hr, and IKKα or IKKβ activity was determined by Western blot analysis with anti-p100/p52 or anti-phospho-IκBα antibody, respectively. Since the promoters of IκBα and p100 contain κB-binding sites and are regulated by the canonical NF-κB pathway [20–22], the protein levels of p100 and IκBα were induced in BL41 cells expressing LMP1 as previously reported (Figure 3A, compare lane 2 with lane 1) [6,9,23]. In DMSO treated cells, LMP1 induced p100 processing to p52 and IκBα phosphorylation (Figure 3A, compare lane 2 with lane 1). On the other hand, the CH_2_Cl_2_ fraction blocked LMP1-induced p100 processing and IκBα phosphorylation (Figure 3A, compare lane 4 with lane 2). The protein levels of p100 and IκBα in cells treated with the CH_2_Cl_2_ fraction were decreased due to inactivation of the canonical NF-κB pathway (Figure 3A, compare lanes 3 and 4 with lanes 1 and 2). Furthermore, the CH_2_Cl_2_ fraction significantly attenuated both LPS- and IL-1β-induced IKKβ activation (Figure 3B and C, compare lanes 6 to 10 with lanes 1 to 5). Thus, these data suggest that the CH_2_Cl_2_ fraction of CIE inhibits NF-κB activation possibly by interfering with IKK activation. 

### The effect of the CH_2_Cl_2_ fraction of CIE on LCL viability

**Figure 4:**
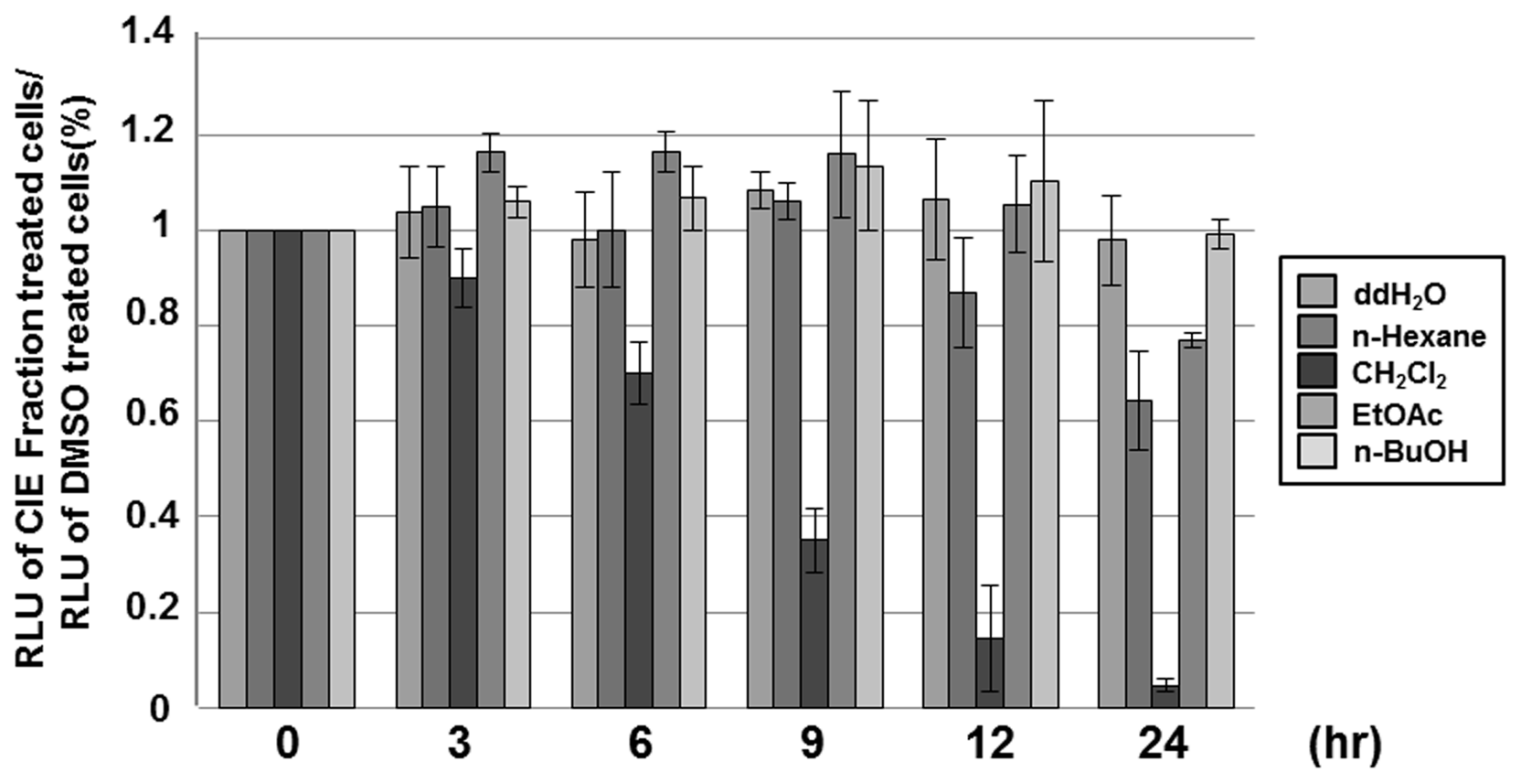
The CH_2_Cl_2_ fraction of CIE reduces LCL viability. LCLs were treated with either DMSO or *n*-Hexane, CH_2_Cl_2_, EtOAc or *n*-BuOH fraction of CIE at 100µg/ml, and cell viability was determined at 0, 3, 6, 9, 12 or 24hr after treatment using CellTiter-Glo Luminescent Cell Viability Assay. A score of 1.0 indicates that there is no difference in viability between DMSO or CIE fraction treated cells. Luciferase data shown here represent three independent experiments.

Since LMP1-induced NF-κB activation is essential for LCL survival, the effect of the CH_2_Cl_2_ fraction of CIE on the viability of LCLs was investigated. LCLs were treated with 100µg/ml of CIE fractions, and the cell viability was measured using the CellTiter-Glo assay at 0, 3, 6, 9, 12 or 24hr after treatment (Figure 4). Consistent with the NF-κB-dependent luciferase reporter data, the CH_2_Cl_2_ fraction strongly reduced LCL viability (Figure 4). After treatment with the CH_2_Cl_2_ fraction, LCL viability was reduced by 65% at 9hr and by 94% at 24hr (Figure 4). Interestingly, other fractions had almost no effect on LCL viability (Figure 4). 

**Figure 5:**
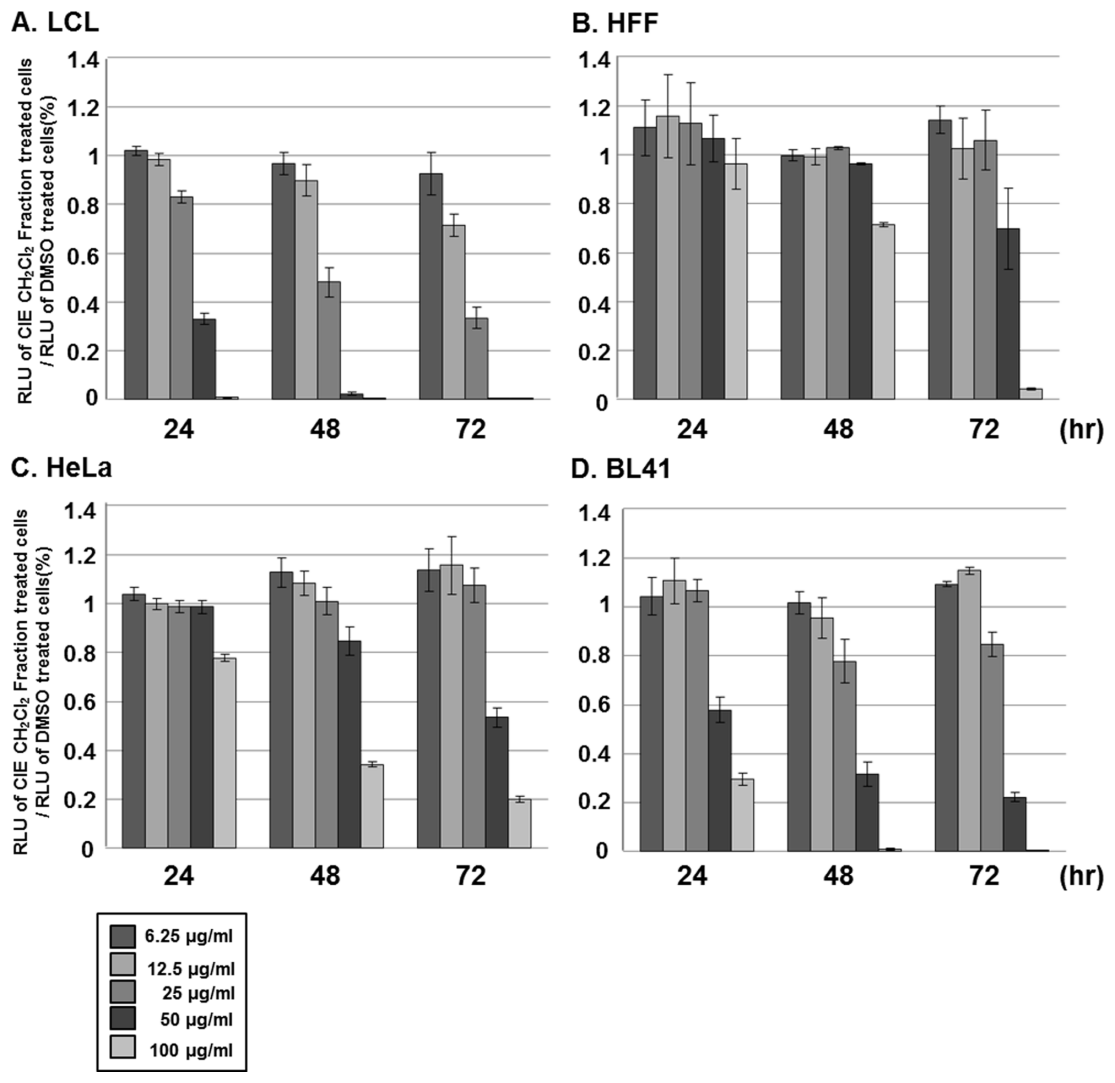
The CH_2_Cl_2_ fraction of CIE is more cytotoxic toward LCLs than HFF, HeLa or BL41 cells. (A) LCLs, (B) HFF, (C) HeLa or (D) BL41 cells were treated with either DMSO or CH_2_Cl_2_ fraction of CIE at 6.25, 12.5, 25, 50 or 100µg/ml, and cell viability was determined at 24, 48 or 72hr after treatment using CellTiter-Glo Luminescent Cell Viability Assay. A score of 1.0 indicates that there is no difference in viability between DMSO or CH_2_Cl_2_ fraction of CIE treated cells. Luciferase data shown here represent three independent experiments.

To further assess the relative toxicity of the CH_2_Cl_2_ fraction in other cell types whose survival is independent of NF-κB activation, LCLs, HFF, HeLa or BL41 cells were treated with 6.25, 12.5, 25, 50 or 100µg/ml of the CH_2_Cl_2_ fraction, and the cell viability was measured by using the CellTiter-Glo assay at 24, 48 or 72hr after treatment (Figure 5). The CH_2_Cl_2_ fraction reduced LCL viability in a dose- and time-dependent manner, and the half maximal inhibitory concentration (IC_50_) values for the cytotoxicity of the CH_2_Cl_2_ fraction on LCLs at 24, 48 and 72hr were 97.3, 55.8 and 45.2mM, respectively (Figure 5A and Table 1). Interestingly, the CH_2_Cl_2_ fraction had very little cytotoxic effect on HFF or HeLa cells. Within the first 24hr after treatment, the CH_2_Cl_2_ fraction had no adverse effect on the viability of HFF or HeLa cells (Figure 5B and C). After 72hr treatment with the CH_2_Cl_2_ fraction at 100µg/ml, HFF or HeLa cell viability was reduced by 91% and 80%, respectively (Figure 5B and C). BL41 cells were slightly more sensitive to the CH_2_Cl_2_ fraction than HFF or HeLa cells. After 24hr treatment with the CH_2_Cl_2_ fraction at 100µg/ml, BL41 cell viability was reduced by 72% and 98% (Figure 5D). Nonetheless, the CH_2_Cl_2_ fraction was evidently less cytotoxic to HFF, HeLa or BL41 cells with IC_50_ values of 145.5, 109.7 and 91.4mM, respectively, at 72hr after treatment (Table 1). 

**Table 1:** The IC_50_ value for the cytotoxicity of the CH_2_Cl_2_ fraction of CIE.

Cell	IC_50_(mM)		
	24hr	48hr	72hr
LCL	97.3	55.8	45.2
HFF	ND^*^	ND^*^	145.5
HeLa	ND^*^	84.3	109.7
BL41	150.9	93.7	91.4

* not determined

**Figure 6:**
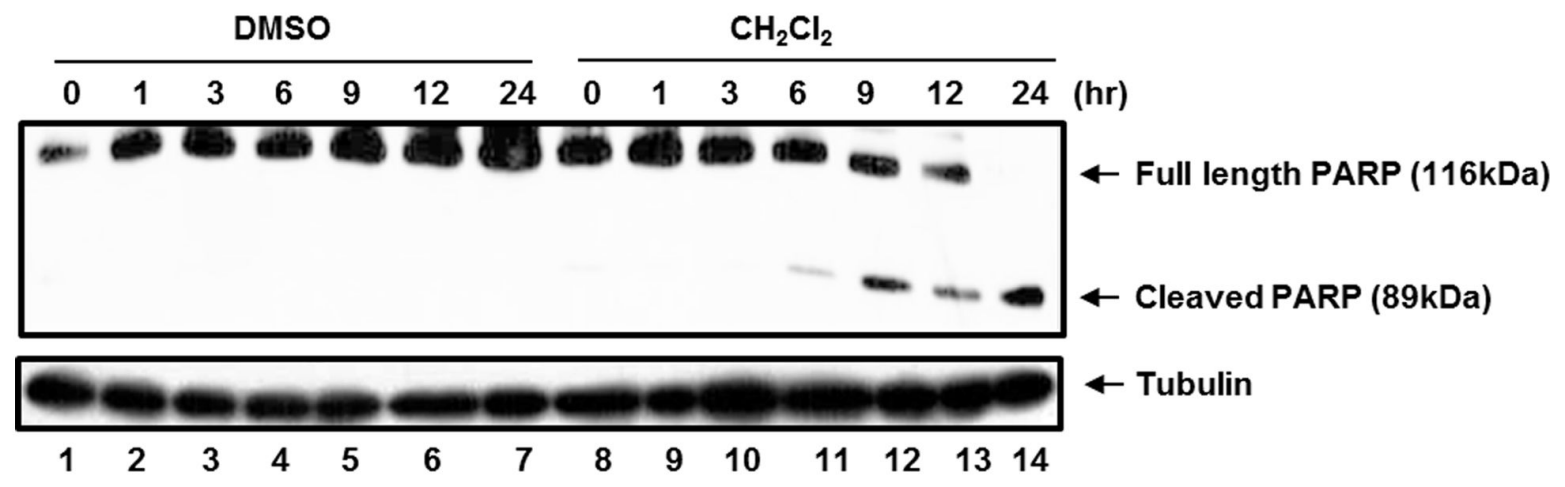
The CH_2_Cl_2_ fraction of CIE induces apoptosis in LCLs. LCLs were treated with 100µg/ml of either DMSO or the CH_2_Cl_2_ fraction of CIE, and PARP cleavage was determined by Western blot analysis at 0, 1, 3, 6, 9, 12 or 24hr after treatment.

Since NF-κB inhibition induces apoptosis in LCLs [9,10], the apoptotic effect of the CH_2_Cl_2_ fraction of CIE in LCL was further assessed. LCLs were treated with 100µg/ml of either DMSO or the CH_2_Cl_2_ fraction, and poly (ADP-ribose) polymerase (PARP) cleavage was determined by Western blot analysis at 0, 1, 3, 6, 9, 12 or 24hr after treatment (Figure 6). At 6hr after treatment, the CH_2_Cl_2_ fraction induced the PARP cleavage (Figure 6, lane 11). At later time points, the PARP cleavage was further induced in cells treated with the CH_2_Cl_2_ fraction (Figure 6, compare lanes 11 to 14 with lanes 4 to 7). Taken together, the CH_2_Cl_2_ fraction of CIE is more cytotoxic toward NF-κB-dependent LCLs than NF-κB-independent HFF, HeLa or BL41 cells. The CH_2_Cl_2_ fraction of CIE may reduce LCL viability by inducing apoptosis.

### The effect of lupeol isolated from CIE on LMP1-induced NF-κB activation

**Figure 7:**
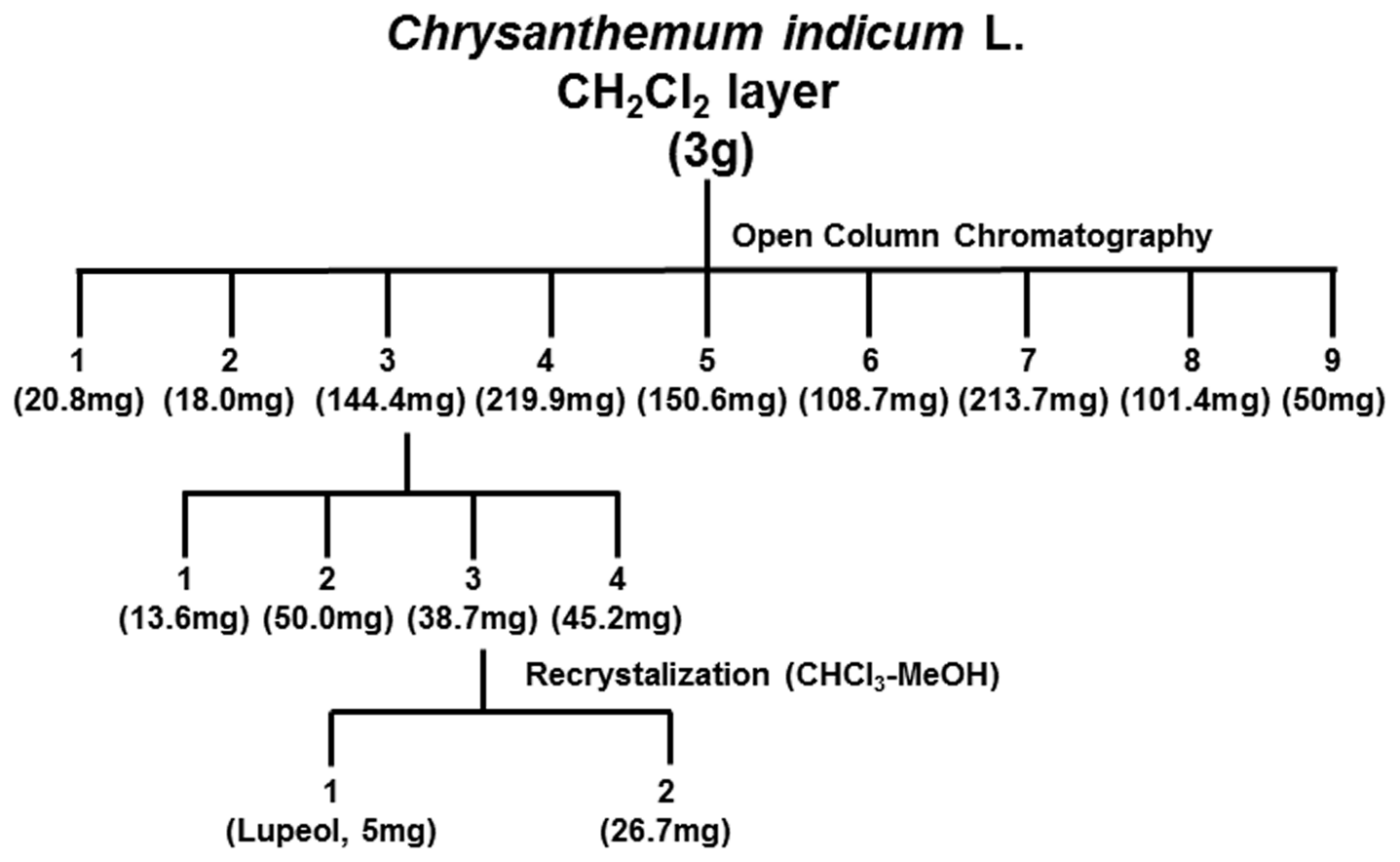
Sub-fractionation and isolation scheme for lupeol from the CH_2_Cl_2_ fraction of CIE.

**Figure 8:**
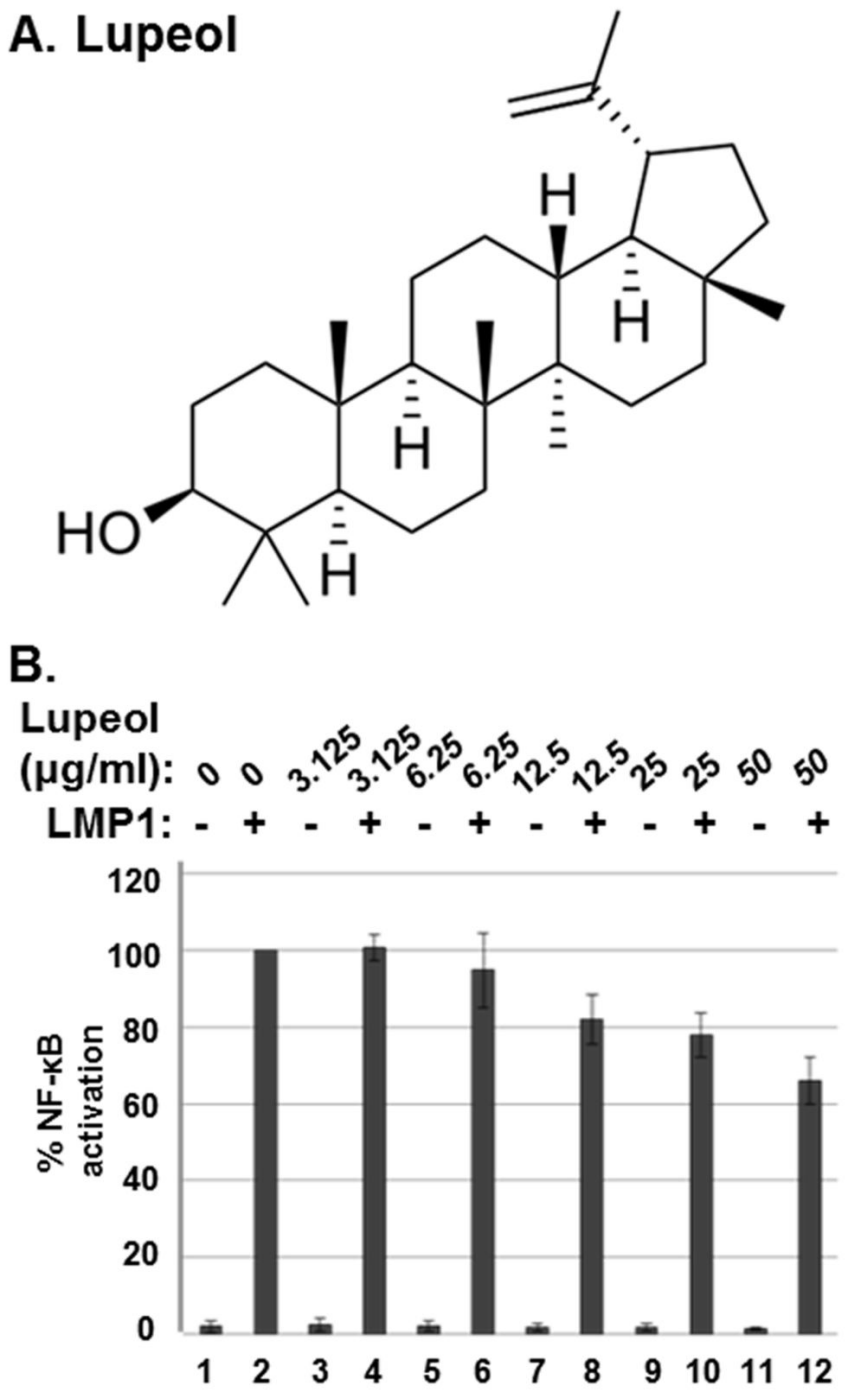
Lupeol inhibits LMP1-induced NF-κB activation. (A) The lupeol structure. (B) HEK293 cells were co-transfected with pSG5 (lanes 1, 3, 5, 7, 9, and 11) or pSG5-FLAG-LMP1 (lanes 2, 4, 6, 8, 10 and 12) plus NF-κB dependent firefly luciferase and control *Renilla* luciferase plasmids. Cells were treated with lupeol at 0, 3.125, 6.25, 12.5, 25 or 50µg/ml, and luciferase activity was measured using a dual luciferase assay system. NF-κB dependent luciferase activity was expressed in RLU by normalizing firefly luciferase activity with constitutive *Renilla* luciferase activity. To calculate relative luciferase activity, LMP1-induced luciferase activities in the presence of lupeol at 0µg/ml was set 100%. Luciferase data shown here represent three independent experiments.

To identify active compounds, the CH_2_Cl_2_ fraction of CIE was further fractionated as described in the Materials and Methods (Figure 7). By performing NF-κB inhibitory activity-guided fractionation, lupeol, a pentacyclic triterpene, was isolated from the CH_2_Cl_2_ fraction of CIE (Figure 8A). To further determine the effect of lupeol on LMP1-induced NF-κB activation, HEK293 cells were co-transfected with pSG5 or pSG5-FLAG-LMP1 plus NF-κB dependent firefly luciferase and control *Renilla* luciferase plasmids and treated with lupeol at 0, 3.125, 6.25, 12.5, 25 or 50µg/ml (Figure 8B). At 50µg/ml, lupeol reduced LMP1-induced NF-κB activation by 34% (Figure 8B, compare lane 12 with lane 1). Thus, lupeol possesses inhibitory activity against LMP1-induced NF-κB activation. Compared to the CH_2_Cl_2_ fraction of CIE which reduced LMP1-induced NF-κB activation by 62%, lupeol was less effective to inhibit LMP1-induced NF-κB activation.

### The effect of lupeol isolated from CIE on LCL viability

**Figure 9:**
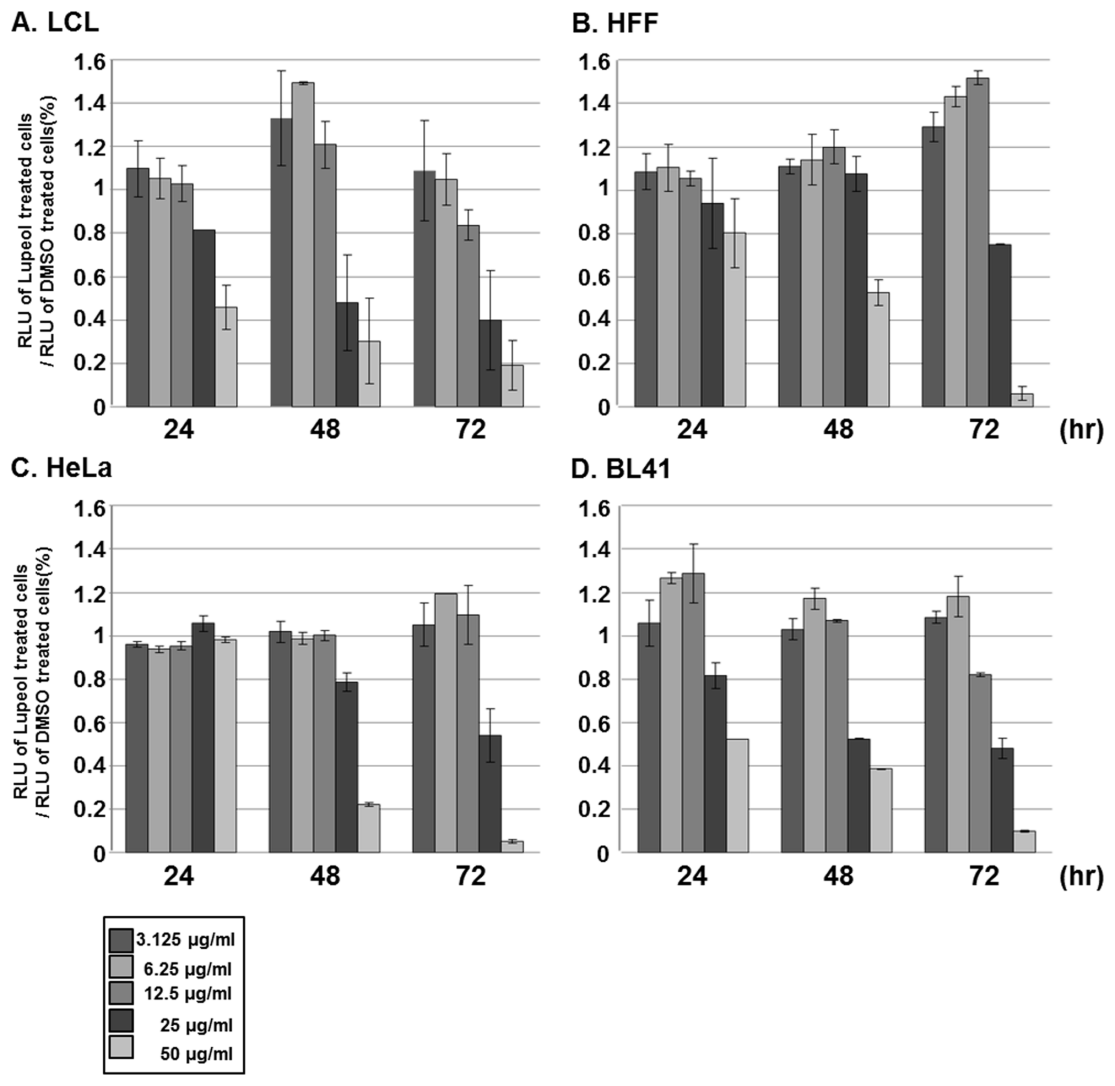
Lupeol is more cytotoxic toward LCLs than HFF, HeLa or BL41 cells. (A) LCLs, (B) HFF, (C) HeLa or (D) BL41 cells were treated with either DMSO or lupeol at 3.125, 6.25, 12.5, 25 or 50µg/ml, and cell viability was determined at 0, 3, 6, 9, 12, 24, 48 or 72hr after treatment using CellTiter-Glo Luminescent Cell Viability Assay. A score of 1.0 indicates that there is no difference in viability between DMSO or lupeol treated cells.

To assess the relative toxicity of lupeol in different cell types, LCLs, HFF, HeLa or BL41 cells were treated with either DMSO or lupeol at 3.125, 6.25, 12.5, 25 or 50µg/ml, and the cell viability was measured by using the CellTiter-Glo assay at 0, 3, 6, 9, 12, 24, 48 or 72hr after treatment (Figure 9). Although lupeol reduced the viability of these cells in a dose- and time-dependent manner at latter time points, it was more cytotoxic toward LCLs than HFF, HeLa or BL41 cells (Figure 9). Within the first 24hr after treatment at 50µg/ml, lupeol reduced LCL viability by 54% (Figure 9A). The IC_50_ values for the cytotoxicity of lupeol on LCLs at 24, 48 and 72hr were 109.9, 57.6 and 51.8mM, respectively (Table 2). On the other hand, lupeol had very little adverse effect on the viability of HFF or HeLa cells within the first 24hr after treatment (Figure 9B and C). At 50µg/ml, lupeol reduced the viability of HFF cells by 48% and 93% at 48 and 72hr after treatment, respectively (Figure 9B). At the same concentration, lupeol reduced the viability of HeLa cells by 78% and 96% at 48 and 72hr after treatment (Figure 9C). BL41 cells were more sensitive to lupeol than HFF and HeLa cells. Within the first 24hr after treatment at 50µg/ml, lupeol reduced the viability of BL41 cells by 48% (Figure 9D). At 48 and 72hr after treatment, 50µg/ml of lupeol further reduced the viability of BL41 cells by 61% and 92%, respectively (Figure 9D). Nonetheless, lupeol was slightly more effective to reduce the viability of LCLs than BL41 cells. The IC_50_ value for the cytotoxicity of lupeol on HFF, HeLa and BL41 cells at 72hr after treatment was 79.7, 63.3 and 56.9mM, respectively (Table 2). 

**Table 2:** The IC_50_ value for the cytotoxicity of lupeol.

Cell	IC_50_(mM)		
	24hr	48hr	72hr
LCL	109.9	57.6	51.8
HFF	ND^*^	ND^*^	79.7
HeLa	ND^*^	88.3	63.3
BL41	ND^*^	68.4	56.9

* not determined

**Figure 10:**
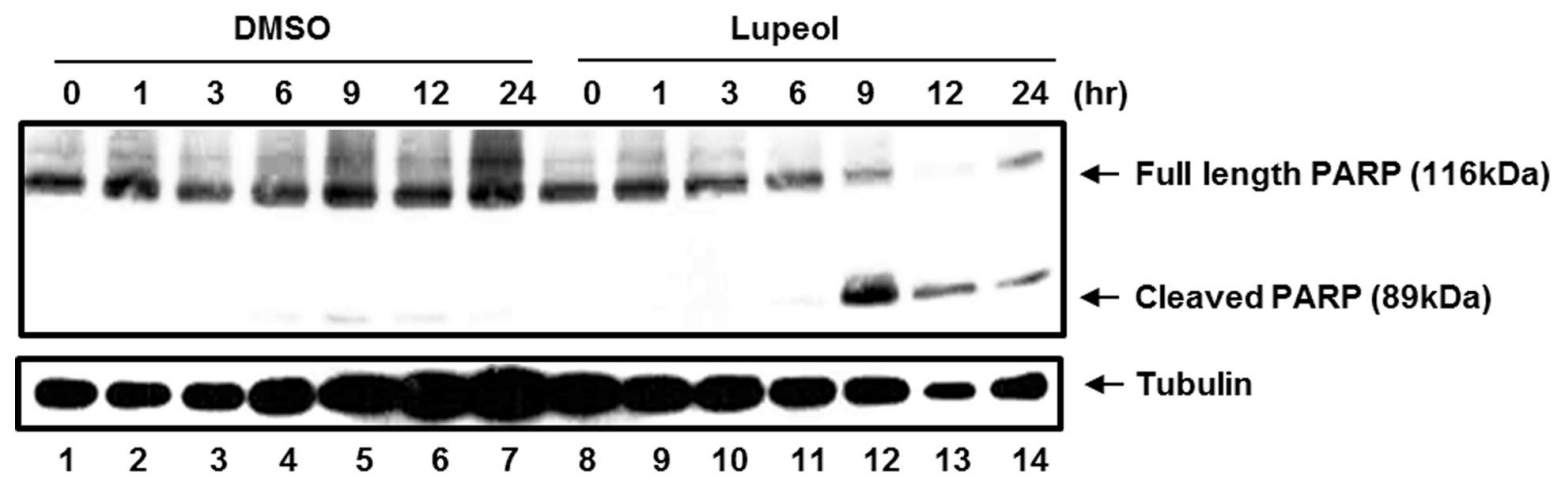
Lupeol induces apoptosis in LCLs. LCLs were treated with 50µg/ml of either DMSO or lupeol, and PARP cleavage was determined by Western blot analysis at 0, 1, 3, 6, 9, 12 or 24hr after treatment.

Furthermore, the apoptotic effect of lupeol in LCL was determined. LCLs were treated with 50µg/ml of lupeol, and PARP cleavage was determined by Western blot analysis at 0, 1, 3, 6, 9, 12 or 24hr after treatment (Figure 10). At 9hr after treatment, lupeol induced the PARP cleavage (Figure 9, lane 12). These data indicate that lupeol induces apoptosis in LCLs and is more cytotoxic to LCLs than HFF, HeLa or BL41 cells. 

## Discussion

The NF-κB family of transcription factors plays an important role in inflammation-associated tumorigenesis [4]. Constitutive activation of NF-κB induced by either mutation in components of the NF-κB pathway or proinflammatory stimuli in the microenvironment has been proposed to promote tumorigenesis [2,24]. Anti-apoptotic function of NF-κB is a major contributor to the survival of numerous cancer cells [24]. NF-κB induces expression of anti-apoptotic genes including caspase-8/FAS-associated death domain (FADD)-like IL-1β-converting enzyme (FLICE) inhibitory protein (c-FLIP), cellular inhibitor of apoptosis (cIAPs) and Bcl2 family proteins such as A1/BFL1 and Bcl-xL [2,25]. Thus, cancer cells in which NF-κB is constitutively activated are resistance to chemo- and radiation therapies [26,27]. In addition, these genotoxic anti-cancer therapies may be ineffective against cancer cells because genotoxic stress induces NF-κB activation [28]. 

NF-κB is also activated in lymphoid cancers associated with tumor viruses such as EBV, Kaposi Sarcoma-associated herpesvirus (KSHV) and human T-cell lymphoma virus (HTLV). These tumor viruses encode proteins that induce NF-κB activation which may contribute to lymphomagenesis [24,29]. Indeed, EBV LMP1 changes the growth phenotype of human B lymphocytes and induces B cell lymphoma when expressed in transgenic mice [30–32]. Since LMP1-induced NF-κB activation is critical for EBV-transformed LCL survival [9,10], LMP1-induced NF-κB activation pathway may be an ideal target for the development of novel therapeutic strategies to treat EBV-associated malignancies. 

We have previously reported that CIE strongly reduces EBV-transformed LCL viability by inhibiting LMP1-induced NF-κB activation [19]. CIE had almost no adverse effects on the viability of cells in which NF-κB is not activated [19]. *C. indicum* has been used to treat inflammatory disease in traditional Korean and Chinese medicine [33–40]. CIE inhibits LPS-induced production of inflammatory cytokines possibly by down-regulating NF-κB and MAPK in RAW264.7 cells macrophages [41]. In addition, CIE inhibits LMP1- and IL-1β-induced NF-κB activation by blocking IKK activity [19]. How CIE inhibits IKK is unclear and is the subject of future studies. CIE may inhibit IKK activation directly by targeting IKK and/or indirectly by blocking the function of components upstream of IKK.

By performing NF-κB inhibitory activity-guided fractionation, we found that the CH_2_Cl_2_ fraction of CIE strongly reduces LMP1-induced NF-κB activation and LCL survival without adversely affecting the viability of HFF, HeLa or BL41 cells. The CH_2_Cl_2_ fraction inhibited LMP1-, IL-1β- and LPS-induced IKK activation. Furthermore, lupeol, a pentacyclic triterpene, was identified in the CH_2_Cl_2_ fraction of CIE to inhibit LMP1-induced NF-κB activation. Lupeol is relatively more cytotoxic toward LCLs in which NF-κB is constitutively activated than HFF, HeLa or BL41 cells. Indeed, lupeol has been reported to inhibit NF-κB activation and induces apoptosis in human cancer cells [42–48]. 

In addition to NF-κB, lupeol affects various cellular signal transduction pathways including Wnt/β-catenin and Akt/protein kinase B (PKB) [44,48,49]. Since the NF-κB, Akt/PKB and Wnt/β-catenin pathways are functionally inter-connected [50–54], lupeol may target a protein(s) commonly utilized by these pathways. At 72hr after treatment, 50µg/ml of lupeol may induce NF-κB-independent cell death in HFF, HeLa or BL41 cells possibly by down-regulating Akt/PKB and/or Wnt/β-catenin pathways. Lupeol may induce both NF-κB-dependent and -independent cell death pathways in LCLs. Thus, the viability of LCLs was relatively more susceptible to lupeol than other cells. 

The CH_2_Cl_2_ fraction of CIE was more effective to inhibit LMP1-induced NF-κB activation and selectively reduce LCL viability than lupeol. Lupeol may interact synergistically with unknown compounds in the CH_2_Cl_2_ fraction of CIE to reduce LMP1-induced NF-κB activation and LCL viability. In addition to the CH_2_Cl_2_ fraction of CIE, the EtOAc or *n*-BuOH fraction also reduced LMP1-induced NF-κB activation by 35% or 31%, respectively. Thus, these fractions may contain additional active compounds that contribute NF-κB inhibitory activity of CIE.

## Materials and Methods

### Plant material and fractionation

The plants materials and 70% ethanol extracts used in this study were collected from Jeju island in Korea through Korea National Research Resource Center (KNRRC, Medicinal Plants Resources Bank No. 2011-0000538) supported by the Korea Research Foundation, where resources were provided by the Ministry of Education, Science and Technology in 2011. The voucher specimens for the samples (specimen number MPRB-KR-04-00034) were deposited at the herbarium of Department of Life Science, Gachon University (GCU).

### Fractionation and isolation of active compounds in CIE

The dried *Chrysanthemum indicum* Linne (1.2kg) was exhaustively extracted by 70% EtOH. The solvent was then evaporated under reduced pressure, at a temperature not exceeding 40°C, to yield 513 g of a semisolid dark yellow residue. The extract was re-suspended in distilled water and successively fractionated with *n*-Hexane, dichloromethane (CH_2_Cl_2_), ethyl acetate (EtOAc), *n*-butanol (n-BuOH) and the ddH_2_O. (Figure 1)

The CH_2_Cl_2_ layer (3g) was chromatographed on a column of Silica (2.5 x 20 cm). Elution was carried out with ddH_2_O followed by 10% stepwise addition of methanol till 100% to give 9 sub-fractions. The 3rd sub-fraction was re-chromatographed on a column of sephadex LH-20. Elution was carried out with CH_2_Cl_2_:ddH_2_O:MeOH = 1:1:3, 1:1:2 and 1:1:1 and then an active compound was isolated by recycling HPLC with solvent, CH_3_Cl-MeOH (Figure 6).

### Identification of lupeol

^1^H-NMR (CDCl_3_, 500 MHz) δ: 0.76 (3H, s, H-28), 0.78 (3H, s, H-23), 0.83 (3H, s, H-25), 0.94 (3H, s, H-24), 0.96 (3H, s, H-27), 1.03 (3H, s, H-26), 1.68 (3H, s, H-30), ), 2.37 (1H, dd, *J* = 11.3, 5.1 Hz, H-19), 3.18 (1H, dd, *J* = 11.3, 5.1 Hz, H-3), ), 4.57 (1H, br s, H-29α), , 4.69 (1H, br s, H-29β); ^13^C-NMR (CDCl_3_, 125 MHz) δ: 14.5 (C-27), 15.3 (C-24), 15.9 (C-26), 16.1 (C-25), 18.0 (C-28), 18.3 (C-6), 19.3 (C-30), 20.9 (C-11), 25.1 (C-12), 27.4 (C-15), 27.4 (C-2), 28.0 (C-23), 29.8 (C-21), 34.3 (C-7), 35.6 (C-16), 37.1 (C-10), 38.0 (C-13), 38.7 (C-1), 38.8 (C-4), 40.0 (C-22), 40.8 (C-8), 42.8 (C-14), 43.0 (C-17), 47.9 (C-18), 48.3 (C-19), 50.4 (C-9), 55.3 (C-5), 79.0 (C-3), 109.3 (C-29), 150.9 (C-20) (Figure 7A).

### Cells, plasmids, reagents and transfections

Primary human foreskin fibroblast (HFF), HEK293, HeLa and mouse leukemic monocyte-macrophage Raw 264.7 cells were maintained in Dulbecco’s modified Eagle’s medium (Biowest, Nuaille, France) supplemented with 10% fetal bovine serum (Biowest) and penicillin (100U/ml) and streptomycin (100µg/ml). Burkitt’s lymphoma BL41 cells were maintained in RPMI 1640 (Life Technologies, Carlsbad, CA) medium supplemented with 10% fetal bovine serum (Biowest) and penicillin (100U/ml) and streptomycin (100µg/ml). IB4, an EBV-infected cord blood derived LCLs and BL41 cells in which LMP1 expression can be negatively controlled by Doxycycline (Dox) were kindly provided by Dr. Elliott Kieff (Harvard Medical School) [9,55]. The pcDNA3FLAG-LMP1 was previously described [56]. Recombinant human IL-1β and LPS from E. Coli Serogroup 0111:B4 were purchased from R&D Systems (Minneapolis, MN) and InvivoGen (San Diego, CA), respectively. Effectene for transient transfection was used according to the manufacturer’s directions (Qiagen, Valencia, CA). 

### Western blot analysis

Cells were collected, fractionated, and transferred to nitrocellulose membranes as described previously [57]. Polyclonal rabbit antibody to p100/p52 was a kind gift from Dr. Ulrich Siebenlist (NIH). Antibodies to phospho-IκBα, IκBα and PARP were purchased from Cell Signaling Technology (Beverly, MA). An antibody to alpha-tubulin was purchased from Sigma Aldrich (St. Louis, MO). Enhanced chemiluminescence detection reagents (Pierce, Rockford, IL) and secondary peroxidase-labeled anti-mouse or anti-rabbit immunoglobulin G antibody (Amersham Biosciences, Piscataway, NJ.) were used according to the manufacturer’s directions.

### NF-κB luciferase reporter and cell viability assays

NF-κB luciferase reporter assay was performed as previously described [58]. Cell viability was determined using CellTiter-Glo luminescent cell viability assay (Promega, WI) according to the manufacturer’s directions. 
